# Supplementary material for: A Scoping Review of the Application of Metabolomics in Nutrition Research: The Literature Survey 2000–2019
Source: Nutrients. 2021 Oct 24;13(11):3760. doi: 10.3390/nu13113760 (PMC8623534; doi:10.3390/nu13113760)
Supplement: Supplementary file 1 [file nutrients-13-03760-s001.zip › nutrients-1414242-supplementary.pdf]

Table S1. Literature research strategy (details)

**Medline Search**	
**Search engine**	PubMed
**Keywords** [1] **Search formula**	(metabolomics OR metabonomics) AND (nutrition OR food OR diet OR meal OR intake OR consumption)
**Species**	Humans
**Publication date**	2000-2019
**Text availability**	Full text
**Publication type**	Excluding: review/systematic review
**Database Search formula**	**((((metabolomics) OR (metabonomics)) AND ((((((nutrition) OR (food)) OR (diet)) OR (meal)) OR (intake)) OR (consumption))) AND (("2000"[Date - Publication] : "2019"[Date - Publication]))) NOT ((review[Publication Type]) OR (systematic review[Publication Type])) Filters: Full text, Humans**
**Transitions**	
metabolomics:	"metabolome"[MeSH Terms] OR "metabolome"[All Fields] OR "metabolomes"[All Fields] OR "metabolomics"[MeSH Terms] OR "metabolomics"[All Fields] OR "metabolomic"[All Fields]
metabonomics:	"metabolomics"[MeSH Terms] OR "metabolomics"[All Fields] OR "metabonomic"[All Fields] OR "metabonomics"[All Fields] OR "metabonome"[All Fields] OR "metabonomes"[All Fields]
nutrition:	"nutrition's"[All Fields] OR "nutritional status"[MeSH Terms] OR ("nutritional"[All Fields] AND "status"[All Fields]) OR "nutritional status"[All Fields] OR "nutrition"[All Fields] OR "nutritional sciences"[MeSH Terms] OR ("nutritional"[All Fields] AND "sciences"[All Fields]) OR "nutritional sciences"[All Fields] OR "nutritional"[All Fields] OR "nutritionals"[All Fields] OR "nutritions"[All Fields] OR "nutritive"[All Fields]
food:	"food"[MeSH Terms] OR "food"[All Fields]
diet:	"diet"[MeSH Terms] OR "diet"[All Fields]
meal:	"meals"[MeSH Terms] OR "meals"[All Fields] OR "meal"[All Fields]
intake:	"intake"[All Fields] OR "intake's"[All Fields] OR "intakes"[All Fields]
consumption:	"consumptions"[All Fields] OR "economics"[MeSH Terms] OR "economics"[All Fields] OR "consumption"[All Fields]

[1] Keywords were converted to PubMed-defined transition terms in the database search formula.

Table S2-1. Classifications – main category

Study design	Biofluid	Application field	Dietary factor
01 RCT parallel-group	01 Blood (plasma/serum)	01 Dietary assessment	01 Nutrient
02 RCT crossover	02 Urine	02 Metabolic profiling	02 Food group
03 Non-randomized trial	03 Feces	03 Risk prediction	03 Dietary pattern
04 Cohort follow-up	04 Saliva	04 Gut microbiota diversity	
05 Case-control	05 Human milk	05 Genetic interaction	
06 Cross-sectional	06 Other	06 Human milk profiling	
07 Case series/other		07 Diet sensitivity	

RCT, randomized controlled trial.

Table S2-2. Classifications – subcategory

Nutrient	Food group	Dietary pattern	Targeted health risk
01 Proteins/amino acids	01 Meat	01 Mediterranean	01 Metabolic syndrome in general
02 Lipids/fatty acids	02 Fish and seafood	02 New Nordic	02 Obesity
03 Carbohydrates/sugars	03 Dairy products	03 HEI/aHEI	03 Hypertension
04 Vitamins/coenzymes	04 Eggs	04 DASH	04 Lipid metabolism/hyperlipidemia
05 Minerals	05 Fruit	05 HDI (WHO healthy)	05 Prediabetes/diabetes
06 Dietary fibers/pre-/probiotics	06 Vegetables	06 Calorie restriction	06 Liver disease
07 Non-nutrients/phytochemicals	07 Legumes/soybeans	07 Western-style/high-fat	07 Kidney disease
08 Nutrients in general	08 Potatoes/other tubers	08 Protein-rich	08 Cardiovascular disease
	09 Cereal/grains	09 Carbohydrate-rich	09 Stroke
	10 Confectionary/soda	10 Wholegrain/low-GI	10 Cancer
	11 Nuts	11 Vegetarian/vegan	11 Inflammatory bowel disease
	12 Spices/herbs/condiments	12 Low-carb/ketogenic	12 Bone and muscle/exercise
	13 Coffee/cocoa/tea	13 Fasting	13 Food allergies/celiac disease
	14 Alcohol	14 Gluten-free	14 Aging
	15 Human/formula milk	15 Undernutrition	15 Malnutrition
	16 Multiple food groups	16 Breakfast type	16 Mental health/sensory effects
	17 Other	17 Region	17 Maternal/pediatric health
		18 Other	18 Genetic factors
			19 Other

HEI/aHEI, Healthy Eating Index/alternate Healthy Eating Index. DASH, Dietary Approaches to Stop Hypertension. HDI, Health Diet Indicator. GI, Glycemic Index.

Table S3-1. Aggregated results of main category[1]

Main category	2003	2004	2005	2006	2007	2008	2009	2010	2011	2012	2013	2014	2015	2016	2017	2018	2019	Total	
**Total number of articles**	2	2	2	3	3	1	5	10	13	13	31	38	41	37	69	68	114	**452**	
**Study design (n = 456)**																			
01 RCT parallel-group					1		2	4	4	5	9	9	10	12	21	16	33	**126**	**28%**
02 RCT crossover				2	1		1	1	5	5	10	10	10	3	15	21	29	**113**	**25%**
03 Non-randomized trial	2	1	2	1	1	1	1	2	3		8	5	10	8	6	8	9	**70**	**15%**
04 Cohort follow-up												1	2		10	4	7	**24**	**5%**
05 Case-control								1				2	2	3	5	4	11	**28**	**6%**
06 Cross-sectional		1					1	2	2		3	10	6	9	13	13	24	**84**	**18%**
07 Case series/other		1								1	1	1	1	2	1	2	1	**11**	**2%**
**Biofluid (n = 534)**																			
01 Blood	2			2	2		4	4	8	6	19	23	26	24	53	50	77	**300**	**56%**
02 Urine	1	2	2	3	3	1	4	6	7	7	12	13	15	15	28	21	29	**169**	**32%**
03 Feces										1	2	2	5	5	7	3	16	**41**	**8%**
04 Saliva				1								2						**3**	**1%**
05 Human milk								1		1	2	1	1	2		3	6	**17**	**3%**
06 Other															1	2	1	**4**	**1%**
**Application field (n = 452)**																			
01 Dietary assessment		1			1			1	5		4	10	9	10	18	10	22	**91**	**20%**
02 Metabolic profiling	1	1	2	2	1	1	3	5	6	7	18	19	15	16	26	33	35	**191**	**42%**
03 Risk prediction							1	3	1	1	4	5	9	5	16	18	38	**101**	**22%**
04 Gut microbiota diversity										2	2	2	4	2	7		11	**30**	**7%**
05 Genetic interaction									1		1				1	1	3	**7**	**2%**
06 Human milk profiling								1		1	1	1	1	2		3	5	**15**	**3%**
07 Diet sensitivity	1			1	1		1			2	1	1	3	2	1	3		**17**	**4%**
**Dietary factor (n = 428)**																			
01 Nutrient	1		1		1			2	3	1	8	10	11	5	14	17	25	**99**	**23%**
02 Food group		1	1	2	2	1	4	4	10	9	15	18	17	18	35	24	43	**204**	**48%**
03 Dietary pattern		1					1	3		1	7	9	11	10	19	22	41	**125**	**29%**

RCT, randomized controlled trial.

[1] Studies are categorized by the main subject described in the article and are placed in multiple categories when multiple items are the main target.

Table S3-2. Aggregated results of dietary factor[1]

Subcategory	2003	2004	2005	2006	2007	2008	2009	2010	2011	2012	2013	2014	2015	2016	2017	2018	2019	Total	
**Nutrient (n = 100)**																			
01 Proteins/amino acids													1		1	5	3	**10**	**10%**
02 Lipids/fatty acids											3	3	2	2	2	3	10	**25**	**25%**
03 Carbohydrates/sugars										1					1	1		**3**	**3%**
04 Vitamins/coenzymes								1	3		1	1	2	1	6	3	2	**20**	**20%**
05 Minerals									1				2			1	2	**6**	**6%**
06 Dietary fibers/pre-/probiotics								1			3	2			2	1	1	**10**	**10%**
07 Non-nutrients	1		1		1						1	3	4	2	2	2	7	**24**	**24%**
08 Nutrients in general												1				1		**2**	**2%**
**Food group (n = 206)**																			
01 Meat				1	1			1					1	1	3		3	**11**	**5%**
02 Fish and seafood													1	1	3		2	**7**	**3%**
03 Dairy products					1							1	1	1	3	3	5	**15**	**7%**
04 Eggs																	1	**1**	**0%**
05 Fruit								1	1	1	5	3	4	1	6	5	4	**31**	**15%**
06 Vegetables									1		3	1				1	1	**7**	**3%**
07 Legumes/soybeans										1	1				2	3	1	**8**	**4%**
08 Potatoes/other tubers																		**0**	**0%**
09 Cereal/grains									2	1	2	2	2	2	3		2	**16**	**8%**
10 Confectionary/soda					1		1			1			1	1	2			**7**	**3%**
11 Nuts								1	1	2	1	1	2		1		2	**11**	**5%**
12 Spices/herbs/condiments										1							3	**4**	**2%**
13 Coffee/cocoa/tea			1	1		1	3	1	2		1	2	3	2	2	4	5	**28**	**14%**
14 Alcohol		1								2	1	1	1	5	1	5	4	**21**	**10%**
15 Human/formula milk												3		2	5	1	7	**18**	**9%**
16 Multiple food groups									3		1	4	1	2	5	2	3	**21**	**10%**
17 Other																		**0**	**0%**
**Dietary pattern (n = 130)**																			
01 Mediterranean													2	3	6	2	2	**15**	**12%**
02 New Nordic												1		1	1	1	3	**6**	**5%**
03 HEI/aHEI															1	1	3	**3**	**2%**
04 DASH													1		1	1		**4**	**3%**
05 HDI (WHO healthy)															2		1	**2**	**2%**
06 Calorie restriction							1					1	1	1	3	3	1	**11**	**8%**
07 Western-style/high-fat											2	1	1			4	4	**12**	**9%**
08 Protein-rich										1					1		1	**3**	**2%**
09 Carbohydrate-rich																	2	**2**	**2%**
10 Wholegrain/low-GI								1			1	1	2			1	3	**9**	**7%**
11 Vegetarian/vegan								1				1	1	2		2	3	**10**	**8%**
12 Low-carb/ketogenic											1				1		1	**3**	**2%**
13 Fasting											1	1				2	4	**8**	**6%**
14 Gluten-free																	2	**2**	**2%**
15 Undernutrition												1	2	2	1		5	**11**	**8%**
16 Breakfast type											1				1		1	**3**	**2%**
17 Region		1						1				1	1				4	**8**	**6%**
18 Other											1	1		1	4	5	6	**18**	**14%**

HEI/aHEI, Healthy Eating Index/alternate Healthy Eating Index. DASH, Dietary Approaches to Stop Hypertension. HDI, Health Diet Indicator. GI, Glycemic Index.

[1] Studies are categorized by the main subject described in the article and are placed in multiple categories when multiple items are the main target.

Table S3-3. Aggregated results of targeted health risk[1]

Subcategory	2003	2004	2005	2006	2007	2008	2009	2010	2011	2012	2013	2014	2015	2016	2017	2018	2019	Total	
**Targeted health risk (n = 239)**																			
01 Metabolic syndrome in general							1		1	1	1	2	3		3	2	4	**18**	**8%**
02 Obesity											3	3	2	1	4	6	5	**24**	**10%**
03 Hypertension											1		2	1	2	3	1	**10**	**4%**
04 Lipid metabolism/hyperlipidemia																1	1	**2**	**1%**
05 Prediabetes/diabetes								1	1			2	4	4	7	6	12	**37**	**15%**
06 Liver disease								2						1			4	**7**	**3%**
07 Kidney disease																	2	**2**	**1%**
08 Cardiovascular disease								1		1	4	2	3	2	6	7	9	**35**	**15%**
09 Stroke																		**0**	**0%**
10 Cancer							1		1		1	1	4	1	2	1	7	**19**	**8%**
11 Inflammatory bowel disease								1						1	2		4	**8**	**3%**
12 Bone and muscle/exercise									1	1	1					1	2	**6**	**3%**
13 Food allergies/celiac disease										1				1	1		2	**5**	**2%**
14 Aging											1		1		1	1	3	**7**	**3%**
15 Malnutrition												1	1	2	1		2	**7**	**3%**
16 Mental health/sensory effects					1		1			1		2	1	1	1	1	1	**10**	**4%**
17 Maternal/pediatric health								1	1		1	4	1	3	5	4	6	**26**	**11%**
18 Genetic factors															1	1	2	**4**	**2%**
19 Other													1		4	2	5	**12**	**5%**

[1] Studies are categorized by the main subject described in the article and are placed in multiple categories when multiple items are the main target.
